# Tanimilast, A Novel Inhaled Pde4 Inhibitor for the Treatment of Asthma and Chronic Obstructive Pulmonary Disease

**DOI:** 10.3389/fphar.2021.740803

**Published:** 2021-11-23

**Authors:** Fabrizio Facchinetti, Maurizio Civelli, Dave Singh, Alberto Papi, Aida Emirova, Mirco Govoni

**Affiliations:** ^1^ Corporate Pre-clinical R&D, Chiesi, Parma, Italy; ^2^ Medicines Evaluation Unit, Manchester University NHS Foundation Hospital Trust, Manchester, United Kingdom; ^3^ Respiratory Medicine, Department of Translational Medicine, University of Ferrara, Ferrara, Italy; ^4^ Global Clinical Development, Chiesi, Parma, Italy

**Keywords:** phosphodiesterase 4 inhibitors (PDE4i), asthma, COPD—chronic obstructive pulmonary disease, inhaled administration, inflammation

## Abstract

Chronic respiratory diseases are the third leading cause of death, behind cardiovascular diseases and cancer, affecting approximately 550 million of people all over the world. Most of the chronic respiratory diseases are attributable to asthma and chronic obstructive pulmonary disease (COPD) with this latter being the major cause of deaths. Despite differences in etiology and symptoms, a common feature of asthma and COPD is an underlying degree of airways inflammation. The nature and severity of this inflammation might differ between and within different respiratory conditions and pharmacological anti-inflammatory treatments are unlikely to be effective in all patients. A precision medicine approach is needed to selectively target patients to increase the chance of therapeutic success. Inhibitors of the phosphodiesterase 4 (PDE4) enzyme like the oral PDE4 inhibitor roflumilast have shown a potential to reduce inflammatory-mediated processes and the frequency of exacerbations in certain groups of COPD patients with a chronic bronchitis phenotype. However, roflumilast use is dampened by class related side effects as nausea, diarrhea, weight loss and abdominal pain, resulting in both substantial treatment discontinuation in clinical practice and withdrawal from clinical trials. This has prompted the search for PDE4 inhibitors to be given by inhalation to reduce the systemic exposure (and thus optimize the systemic safety) and maximize the therapeutic effect in the lung. Tanimilast (international non-proprietary name of CHF6001) is a novel highly potent and selective inhaled PDE4 inhibitor with proven anti-inflammatory properties in various inflammatory cells, including leukocytes derived from asthma and COPD patients, as well as in experimental rodent models of pulmonary inflammation. Inhaled tanimilast has reached phase III clinical development by showing promising pharmacodynamic results associated with a good tolerability and safety profile, with no evidence of PDE4 inhibitors class-related side effects. In this review we will discuss the main outcomes of preclinical and clinical studies conducted during tanimilast development, with particular emphasis on the characterization of the pharmacodynamic profile that led to the identification of target populations with increased therapeutic potential in inflammatory respiratory diseases.

## Introduction

Phosphodiesterase-4 (PDE4) is a cyclic adenosine 3 monophosphate (cAMP)-specific phosphodiesterase located predominantly in cells involved in inflammation. PDE4 inhibitors by preventing the breakdown of cAMP, have the potential to reduce inflammatory-mediated processes in the context of the pathophysiology of respiratory diseases (Sanz et al., 2005).

Chronic obstructive pulmonary disease is characterized by poorly reversible airflow obstruction accompanied with persistent airway inflammation. Patients with COPD suffer with symptoms including dyspnea, cough and sputum production, and many also experience exacerbations which are a sudden worsening of symptoms that requires additional pharmacological treatment. The Global initiative for Chronic Obstructive Lung Diseases (GOLD, 2021) recommends maintenance use of double and triple inhaled combination treatments that include glucocorticoids (ICS), long-acting β2-agonists (LABA), and long-acting muscarinic-receptor antagonists (LAMA) in order to prevent exacerbations (GOLD, 2021). However, approximately 30–40% of patients are reported to continue to have moderate or severe exacerbations despite being treated with triple inhaled therapy (ICS plus LABA plus LAMA) (Vestbo et al., 2017; Langham et al., 2019). These patients have limited additional add-on treatment options: GOLD suggests azithromycin treatment for former smokers or the oral PDE4 inhibitor roflumilast in patients with a chronic bronchitis phenotype and with forced expiratory volume in 1 s (FEV1) lower than 50% of their predicted value (GOLD, 2021). Indeed, several clinical trials have demonstrated that roflumilast improves lung function and reduces exacerbations frequency in COPD patients, particularly in those with a chronic bronchitis phenotype (Sturton and Fitzgerald, 2002; Calverley et al., 2009; Rennard et al., 2011; Bardin et al., 2015; Martinez et al., 2015, 2016). However, oral administration of roflumilast at therapeutic doses causes a high rate of treatment discontinuation due to gastrointestinal (GI) and other class related side effects such as diarrhea, nausea, decreased appetite, weight loss and psychiatric symptoms (Muñoz-Esquerre et al., 2015; Rogliani et al., 2016). The development of inhaled PDE4 inhibitors have the potential to minimize the adverse events related with the systemic inhibition of PDE4 and thus improve the tolerability of this class of drugs (Phillips, 2020). Tanimilast (international non-proprietary name (INN) of CHF6001) is a novel PDE4 inhibitor that has been specifically designed and formulated to be delivered via inhalation and to have a robust pulmonary anti-inflammatory profile coupled with low systemic exposure and low emetic effects (Moretto et al., 2015) (Villetti et al., 2015). It is available in powder for inhalation administered via NEXThaler^®^, Chiesi proprietary multi-dose inhaler (Mariotti et al., 2018; Singh et al., 2019).

Tanimilast has currently completed a phase II clinical development program (Singh et al., 2020c) and started Phase III as a treatment to reduce the risk of exacerbations in COPD patients with chronic bronchitis and a history of exacerbations, as an add-on to triple therapy. Here we will review the outcomes of the main studies conducted during preclinical and clinical development of tanimilast with a particular focus on the identification of subgroups of patients with increased chances of therapeutic success.

## Phosphodiesterase-4 Enzyme

PDEs form a superfamily of at least 11 intracellular isoenzymes that are involved in the modulation of signal transduction processes via the degradation of cAMP and cyclic guanosine monophosphate (cGMP) (Bender and Beavo, 2006). Type 4 cyclic nucleotide PDEs is a family of cAMP-specific enzymes encoded by four genes (PDE4A, PDE4B, PDE4C, and PDE4D) sharing a highly conserved catalytic domain and abundantly expressed in leukocytes (Maurice et al., 2014). cAMP is a key regulator of inflammation and a decrease in intracellular cAMP levels, following hydrolysis by PDE enzymes, promotes inflammatory responses (Serezani et al., 2008). By increasing intracellular cAMP levels, PDE4 inhibitors show a broad spectrum of anti-inflammatory effects in almost all cells of the immune system. Indeed, PDE4 is a major player in regulating proinflammatory cellular functions, such as proliferation and cytokine secretion, chemotaxis, degranulation, antibody IgE release and generation of lipid mediators (Giembycz and Newton, 2014; Li et al., 2018). PDE4 regulates also the function of several structural cells that control lung functions such as airway smooth muscle, airway epithelium, vascular endothelium and airway sensory nerves (Sturton and Fitzgerald, 2002; Hatzelmann et al., 2010).

## Historical Overview of Phosphodiesterase-4 Inhibitors for Treating Pulmonary Diseases

The only PDE4 inhibitor currently approved for treating pulmonary diseases is the orally administered roflumilast, which reduces exacerbation rates in patients with COPD (Sturton and Fitzgerald, 2002; Calverley et al., 2009; Rennard et al., 2011; Bardin et al., 2015; Martinez et al., 2015, 2016) and shows also evidence of clinical benefits in asthma (Giembycz and Newton, 2015) although it is not labeled for this indication. The clinical dosage and efficacy of roflumilast is limited by class-related side effects, such as nausea, diarrhoea and weight loss that make it intolerable for a significant proportion of patients (Muñoz-Esquerre et al., 2015; Rogliani et al., 2016). Among other PDE4 inhibitors, cilomilast (Ariflo) showed disappointing Phase III clinical results with dose-limiting adverse events probably due to interaction of the compound with PDE4 expressed in “non-target” tissue (Giembycz, 2006). Ensifentrine (RPL554), which is considered a PDE3/4 inhibitor despite its affinity for PDE3 is 3,440 times higher than that for PDE4 (Cazzola et al., 2018), is currently under clinical development for the treatment of asthma and COPD. Different approaches have been pursued for the development of oral PDE4 inhibitors with improved gastrointestinal tolerability. Because emesis is at least in part a side effect mediated by the central nervous system, PDE4 inhibitors with low brain penetration have been developed, but this approach has not yielded better tolerated compounds, given that the area postrema, which acts as chemoreceptor trigger zone for emesis, is not protected by the blood-brain barrier and that PDE4 inhibitors also exert direct effects on the gastrointestinal tract (Okuda et al., 2009). Alternative strategies were developed to selectively target the low-affinity rolipram-binding site conformer of PDE4 over the high-affinity rolipram-binding conformer or the isoform PDE4B over the isoform PDE4D (Rocque et al., 1997), but advantages from both these approaches have not been proven (Souness and Rao, 1997).

Many PDE4 inhibitors have been designed for inhaled administration in respiratory diseases during the last 20 years in the attempt to limit systemic exposure and the associated side effects of PDE4 inhibition. A few of these inhaled PDE4 inhibitors have advanced into clinical trials for treatment of asthma and COPD. As recently reviewed by Phillips (Phillips, 2020), at least four inhaled PDE4 inhibitors have progressed up to phase II, namely, AWD-12-281 (COPD and asthma), Tofimilast (COPD and asthma), UK-500,001 (COPD), GSK256066 (COPD and Asthma). AWD-12-281 and Tofimilast are PDE4 inhibitors with a modest potency and failed to demonstrate efficacy in Phase II clinical trials and their development has been discontinued (Phillips, 2020) (Mealy and Bayés, 2005) (Gutke et al., 2005). UK-500,001 showed lack of clinical activity probably due to its moderate potency and low solubility. In addition, a higher incidence of systemic PDE4 inhibitor-related side effects as diarrhea were observed (Vestbo et al., 2009). GSK256066 is a potent (IC_50_ = 0.003 nM) inhaled PDE4 inhibitors which showed robust anti-inflammatory effects in preclinical animal models of pulmonary inflammation (Tralau-Stewart et al., 2011) (Nials et al., 2011). However, the clinical development was likely hampered by dose limiting safety profile (Watz et al., 2013) (Villetti et al., 2015).

Overall, these past attempts highlight the difficulties in the development of potent and selective inhaled PDE4 inhibitors with optimal target engagement in the lung and limited systemic exposure.

## The Discovery of Tanimilast

**FIGURE 1:**
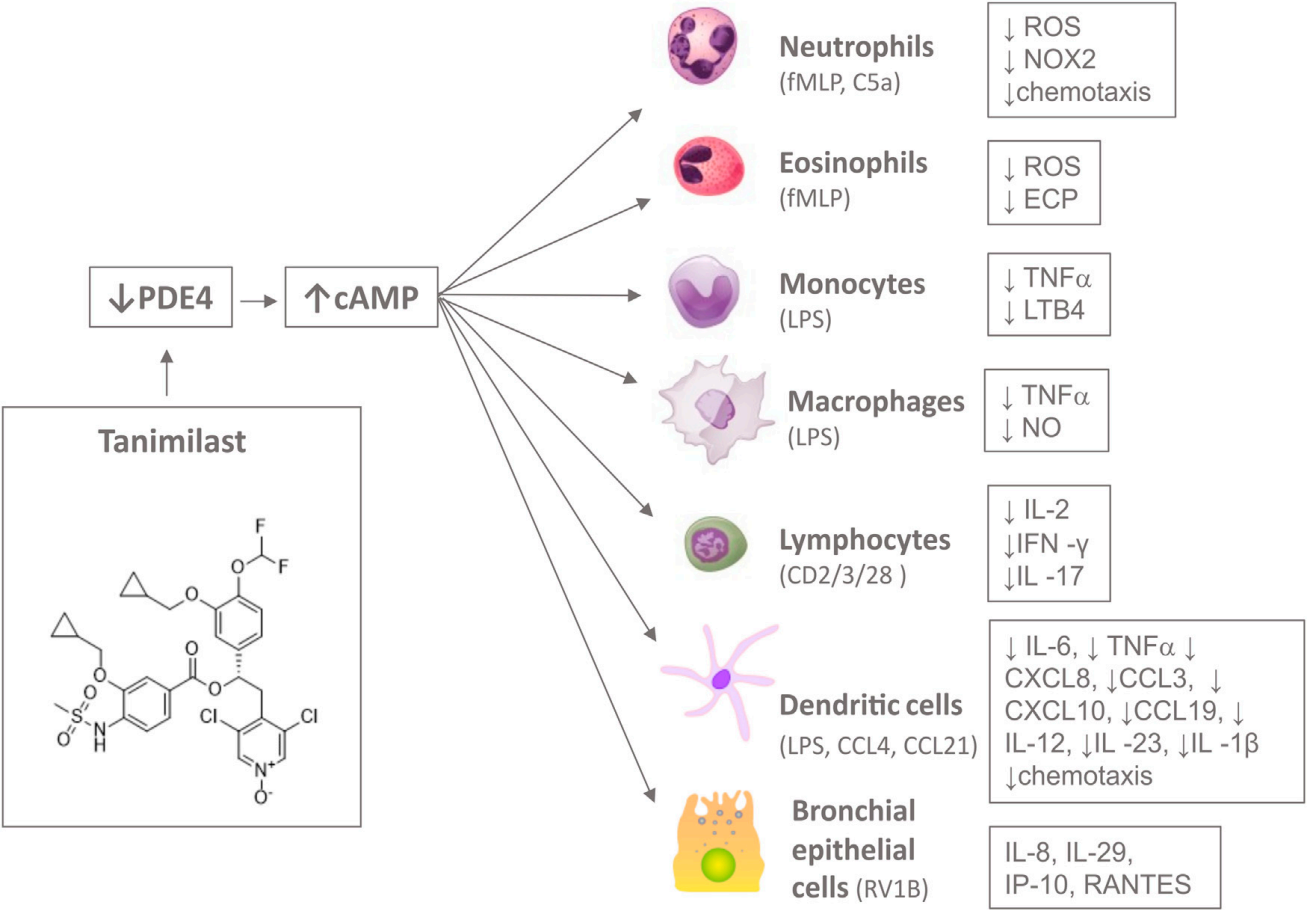
Inhibition of the enzymatic activity of PDE4 by tanimilast induces a rise of the second intracellular messenger cAMP. This results in a decreased release of a wide range of inflammatory mediators and chemotaxis in several cells type of the immune system, including neutrophils, eosinophils, monocytes, macrophages, lymphocytes, dendritic cells and bronchial epithelial cells upon various stimuli (in brackets). Figure objects were adapted by OpenStax College and Database Center for Life Science (DBCLS) CC BY 3.0 https://creativecommons.org/licenses/by/3.0 and !Original: ArcadianVector: XcepticZP, Public domain, via Wikimedia Commons.

The medicinal chemistry strategy that brought to the identification and selection of tanimilast from a series of novel ester derivatives of 1-(3-(cyclopropylmethoxy)-4-(difluoromethoxy)phenyl)-2-(3,5-dichloropyridin-4-yl) ethanol has been described previously in details (Armani et al., 2014). A rational drug design, based on the analysis of the PDE4 catalytic binding pocket, brought to the identification of several compounds with structural modifications of the benzoic moiety to maximize the inhibitory potency against PDE4 enzymatic activity and the anti-inflammatory effects in peripheral blood mononuclear cell-based assays. Several novel PDE4 inhibitors with potent anti-inflammatory activity *in vitro* were identified during the screening campaign. Among the different candidates tanimilast (referred to initially as compound 32a) proved to be the most interesting derivative, displaying the best combination of high potency, prolonged half life in the lung, low permeability, and very high protein plasma binding (PPB), with the latter being important to limit the drug free fraction in the systemic circulation (Armani et al., 2014). Tanimilast was selected also for its ability to make extended interactions with all three regions of the PDE4B catalytic binding pocket, resulting not only in enhanced inhibitory potency but also in favourable binding kinetics (rapid association to PDE4 coupled with very slow dissociation) (Armani et al., 2014). The chemical structure of tanimilast [(S)-3,5-dichloro-4-(2-(3-(cyclopropylmethoxy)-4-(difluoromethoxy)phenyl)-2-(3-(cyclopropylmethoxy)-4-(methylsulfonamido)benzoyloxy)ethyl)pyridine 1-oxide] is shown in Figure 1 (Moretto et al., 2015).

## Preclinical Overview

### On Target Pharmacology and Selectivity

Tanimilast was about 10- and 900-fold more potent than roflumilast and cilomilast, respectively, in inhibiting PDE4 enzymatic activity (IC_50_ = 0.026 ± 0.006 nM), with the latter being a discontinued orally active PDE4 inhibitor developed up to Phase 3 in COPD (Giembycz, 2006). Tanimilast inhibited PDE4 isoforms A-D with equal potency showing no relevant isoform selectivity, similarly to roflumilast, GSK256066 and UK-500,001 (Armani et al., 2014).

Tanimilast proved to be highly selective versus PDE4 (>20,000-fold selectivity) when challenged against a wide panel of PDEs, including PDE1, PDE2, PDE3, PDE5, PDE6, PDE7A, PDE8A1, PDE9A2, PDE10A2, and PDE11A4 (Moretto et al., 2015).

### Anti-Inflammatory Effects *In-Vitro*

Tanimilast was tested for its anti-inflammatory activity in several assays based on different human cell types including dendritic cells, neutrophils, eosinophils, macrophages, lymphocytes and bronchial epithelial cells as summarized in Figure 1 and Table 1.

**TABLE 1:** Tanimilast anti-inflammatory effects *in vitro* in various cell-based assays encompassing human and rodents’ cellular preparations.

Cell/tissue	Source	Stimuli	Tanimilast effects	References
Dendritic cells	Human dendritic cells differentiated from peripheral blood monocytes (moDCs)	Chemotactic agents (CCL4, CCL21, chemerin), LPS	Decreased chemotaxis to CCL4, CCL21 and chemerin. Decreased the release of pro-inflammatory cytokines (TNF-α and IL-6), chemokines (CXCL8, CCL3, CXCL10 and CCL19) and of Th1- and Th17-polarizing cytokines (IL-12, IL-23 and IL-1β)	Gianello et al. (2019)
Neutrophils	Human peripheral blood healthy subjects	fMLP/cytochalasin, influenza virus, C5a-induced chemotaxis	Inhibited reactive oxygen species (ROS) release, NOX2 and chemotaxis	Moretto et al. (2015)
	Sabogal Piñeros et al. (2020)		
Eosinophils	Human peripheral blood healthy subjects	fMLP/cytochalasin	Inhibited reactive oxygen species (ROS) and eosinophil cationic protein (ECP) release	Moretto et al. (2015)
	Sabogal Piñeros et al. (2020)		
Lymphocytes	BALF from mild and moderate asthmatics	T-cell activation anti CD2/3/28 beads	Inhibited IFN-γ, IL-2 and IL-17 release	Southworth et al. (2019)
PBMC	Peripheral blood healthy subjects	LPS	Reduced TNF-α release	Moretto et al. (2015)
Human blood cells	Human “buffy coat”	LPS (1 μg/ml)	Inhibited LTB4 production	Stellari et al. (2019)
Macrophagic cell lines	Raw264.7 (mouse) NR8383 (rat) THP-1 (human)	LPS	Reduced TNF-α and NO release	Moretto et al. (2015)
Macrophages	Isolated from resected lung tissue of COPD patients	LPS	Reduced TNF-α release	Lea et al. (2019)
Lung tissue explants	Resected lung tissue of COPD patients	LPS	Reduced TNF-α release	Lea et al. (2019)
Precision-cut lung slices	Guinea pig	Methacholine	Inhibited methacholine-induced TGF-β release, smooth muscle-myosin increase and airways remodelling	Kistemaker et al., 2017
Human bronchial epithelial cells	BEAS2B cell line	Rhinovirus RV1B	Reduced RV1B-induced upregulation of CXCL8, IL-29, IP-10, and RANTES mRNA and protein. Additive effect to fluticasone	Edwards et al. (2016)

#### Blood Leukocytes and Macrophagic Cells

Tanimilast showed sub-nanomolar potency for inhibition of the release of the pro-inflammatory cytokine TNF-α from peripheral blood mononuclear cells (PBMC) stimulated with LPS (Moretto et al., 2015). The compound was about 6000-fold more potent than cilomilast for inhibition of interferon-gamma (IFN-γ) release from CD4^+^ T cells. This effect could be relevant to asthma exacerbations, where the inhibition of IFN-γ could be beneficial (Kumar et al., 2006).

Tanimilast was also tested in THP-1 monocytic-derived macrophages and two macrophagic cell lines, namely RAW264.7 which is derived from peritoneal mouse macrophages, and NR8383 which is derived from rat alveolar macrophages. Again, the anti-inflammatory effects of tanimilast were evident in all macrophagic cell lines as TNF-α and nitric oxide release was inhibited with potencies slightly inferior (2- to 10-fold potency difference) to those observed in PBMCs (Moretto et al., 2015), a difference probably reflecting dissimilar PDE4 levels of expression between macrophages and monocytes (Shepherd et al., 2004).

Tanimilast was extremely potent in inhibiting eosinophil activation (Moretto et al., 2015; Sabogal Piñeros et al., 2020), a finding consistent with the notion that PDE4 is prominently expressed in eosinophils, which are key players in shaping the pathogenesis of asthma, and also appear to play a role in a subgroup of COPD patients (Agusti et al., 2018). Neutrophil-dominant pulmonary inflammation is an important feature of COPD and plays a role in non-type-2 asthma poorly responding to corticosteroids (Barnes, 2016, 2019). Tanimilast was highly potent in inhibiting fMLP-evoked ROS production from human neutrophils and C5a-induced chemotaxis in mouse neutrophils (Moretto et al., 2015). Thus, tanimilast targeted neutrophils both through direct inhibition of oxidative burst and chemotaxis, two neutrophilic responses recognized to be scarcely sensitive to glucocorticoids (Hakim et al., 2012).

#### Dendritic Cells

Dendritic cells (DCs) are professional antigen presenting cells responsible for antigen presentation to T naïve cells and activation of adaptive immunity (Soumelis et al., 2007). However, DCs also play a crucial role in inflammation and in shaping pathogen-suited adaptive responses, by releasing pro-inflammatory cytokines and soluble mediators that polarize T effector cells (Sozzani, 2005). When tested *in vitro* on human DCs, tanimilast decreased the release of pro-inflammatory cytokines (TNF-α and IL-6), chemokines (CXCL8, CCL3, CXCL10 and CCL19) and of Th1- and Th17-polarizing cytokines (IL-12, IL-23 and IL-1β) (Gianello et al., 2019). The immune-modulatory effects of tanimilast found in DC could be useful to control Th1/Th17-polarized inflammatory diseases such as COPD and autoimmune inflammatory disorders.

#### Lymphocytes From Asthma Patients

Lymphocytes play a key role in asthma pathophysiology, secreting various cytokines involved in chronic inflammation (Kaur et al., 2012).

Tanimilast suppressed T-cell receptor-stimulated IFN-γ, IL-2 and IL-17 release in BAL cells from both mild and moderate asthma patients (Southworth et al., 2019). Furthermore, the effect of tanimilast on IFN-γ and IL-2 was greater than the corticosteroid 17-beclometasone monopropionate (Southworth et al., 2019). Since tanimilast had an effect greater than corticosteroids on Th1 cytokines from TCR-stimulated BAL cells, it can be suggested that there is therapeutic potential for this inhaled agent in severe asthma patients with increased airway levels of IFNγ which may be an indicator of significant Th1 inflammation and/or might reflect an ongoing anti-viral response (Kumar et al., 2006).

#### Alveolar Macrophages and Lung Tissue From Chronic Obstructive Pulmonary Disease Patients

Increased levels of sputum TNF-α are found during acute exacerbations and the production of TNF-α from human lung tissue appears to be an important regulator for the secretion of other inflammatory cytokines, such as IL-6 and CXCL8 (Chung, 2001; Gerritsen et al., 2005; Fan Chung, 2006). Tanimilast was tested in alveolar macrophages (AM) and lung tissue of COPD patients and healthy controls. Interestingly, mRNA levels of PDE4 subtypes A, B and D were increased in both AM and whole lung tissue of COPD patients compared to healthy controls (Lea et al., 2019). Tanimilast and roflumilast both significantly reduced TNF-α production in AM and whole lung tissue (Lea et al., 2019). Tanimilast was more potent than roflumilast with lower EC_50_s of 0.02, 0.01 and 0.31 nM compared to 0.87, 0.47 and 10.8 nM in respective samples (Lea et al., 2019).

The marked inhibitory effect of tanimilast at low sub-nanomolar concentrations on TNF-α production from COPD AM and lung tissue might, therefore, have important further downstream anti-inflammatory effects on a range of cytokines and chemokines given the role of TNF-α in amplifying the innate immune response.

Tanimilast also inhibited secretion of the chemokines CCL2 (MCP-1) and CCL4 (MIP-1β) from macrophages, an effect associated with increased nuclear levels of phosphorylated cAMP response element-binding protein (CREB), a transcription factor that is activated in response to intracellular cAMP elevation (Lea et al., 2019).

#### Airway Epithelial Cells Infected With Rhinovirus

Many asthma and COPD exacerbations are triggered by respiratory virus infections, with rhinovirus being a common pathogen. Tanimilast showed inhibitory effects on rhinovirus (RV1B)-induced cytokines (CXCL8, IP-10 and RANTES, mRNA and protein) production *in-vitro* on human bronchial epithelial cells (BEAS-2B) (Edwards et al., 2016). Comparisons with roflumilast showed that tanimilast had greater potency and likely improved anti-inflammatory efficacy (Edwards et al., 2016).

In conjunction with fluticasone propionate (FP), tanimilast resulted in enhanced anti-inflammatory activity compared with equivalent doses of steroid or tanimilast when used alone (Edwards et al., 2016). Such additive effects in combination with FP is suggestive of a steroid sparing effect of tanimilast as well as a potential additive efficacy when administered on top of inhaled steroid therapy. Indeed, additive effects of PDE4 inhibitors on both expression and activation of the glucocorticoid receptor (GR) have been previously reported (Ortiz et al., 2012; Roth et al., 2002). In addition, Moodley et al. (2013) have shown that combined roflumilast and FP treatment enhanced GRE-dependent reporter activity and several GRE responsive anti-inflammatory genes. A similar GRE-enhancing effect could be elicited by tanimilast in combination with an ICS. Thus, conceptually, the high level of inflammation seen in severe COPD with chronic bronchitis phenotype may be more responsive to an inhaled glucocorticoid and a PDE4 inhibitor if used together (Giembycz and Newton, 2015).

### Anti-Inflammatory Effects in Experimental Models of Pulmonary Inflammation

Tanimilast was tested in different experimental *in vivo* rodent models of pulmonary inflammation driven by various stimuli, including the bacterial endotoxin LPS, the classic allergen ovalbumin and tobacco smoke as summarized in Table 2. In addition, tanimilast was tested in experimental animal models for emesis, a typical side effect of PDE4 inhibitors. Testing tanimilast in a wide range of preclinical models helped in determining the pharmacological doses to be translated into man and the potential therapeutic index of this drug.

**TABLE 2:** Anti-inflammatory effects of tanimilast in various experimental rodent models of pulmonary inflammation.

Animal model	Main endpoints	Route of tanimilast administration	Tanimilast effects	References
LPS-induced lung inflammation in rats	Neutrophilic lung inflammation	Intratracheal instillation	Inhibition of pulmonary neutrophilia, which reached a statistical significance at 0.1 mmol/kg and was maximal at 1 mmol/kg (77%) similarly to budesonide at 1 mmol/kg (77%)	Moretto et al. (2015)
LPS-induced lung inflammation in mice	NF-κB activation, neutrophilic and cytokines in BALF	Snout-only inhalation of the nebulized drug	Inhibits NF-κB activation, neutrophilic infiltration and cytokines (TNF-α, IL-1b, G-CSF, RANTES) accumulation in BALF	Stellari et al. (2019)
Tobacco smoke-induced lung inflammation in mice	Neutrophilic lung inflammation	Intranasal instillation	Inhibited neutrophil infiltration both upon prophylactic (0.15–0.45 mmol/kg per day) or interventional (0.045–0.45 mmol/kg per day) treatment	Villetti et al. (2015)
Ovalbumin-induced lung eosinophilia in the rat	Eosinophilic lung inflammation and lung function	Intratracheal instillation	Suppressed antigen-induced decline of lung functions, namely, forced vital capacity (FVC) and forced expiratory volume at 200 milliseconds (FEV200), (ED50 = 50.1 mmol/kg) and antigen-induced eosinophilia (ED50 = 50.03 mmol/kg)	Villetti et al. (2015)

In endotoxin (LPS)-exposed rats, tanimilast, when administered by intratracheal instillation at 1 h before and 6 h after LPS challenge, elicited a dose-dependent inhibition of pulmonary neutrophilia, which reached a statistical significance at 0.1 μmol/kg (*p* < 0.05) and was maximal at 1 μmol/kg (77%) similarly to budesonide at 1 mmol/kg (77%) (Moretto et al., 2015). Tanimilast also produced dose-dependent inhibition of inflammatory cell counts in broncho-alveolar lavage fluid (BALF), which reached significance at 0.3 μmol/kg (*p* < 0.01) and was maximal at 1 μmol/kg (61%), similarly to budesonide at 1 μmol/kg (62%) (Moretto et al., 2015).

In an allergen driven rodent model of pulmonary inflammation (ovalbumin-sensitized Brown-Norway rats), intratracheal administration of tanimilast counteracted the antigen-induced decline of lung function (ED_50_ 0.1 μmol/kg) measured as forced vital capacity (FVC) and forced expiratory volume at 200 milliseconds (FEV200) in a forced pulmonary maneuver system provided by Buxco Research Systems (Wilmington, NC) (Villetti et al., 2015). In addition, tanimilast was effective in reducing antigen-induced eosinophilia in BALF (ED_50_ = 0.03 μmol/kg) when administered 24 h before antigen challenge, in agreement with -sustained lung concentrations of tanimilast (up to 72 h) after intratracheal treatment (Villetti et al., 2015).

In mice exposed to 11 days of tobacco smoke, intranasal, once daily administration of tanimilast inhibited neutrophil infiltration both upon prophylactic (0.15–0.45 μmol/kg per day) or interventional (0.045–0.45 mmol/kg per day) treatment (Villetti et al., 2015). Interestingly, neutrophilic inflammation in mice induced by tobacco smoke is resistant to corticosteroids, suggesting that tanimilast is effective also in steroid-resistant inflammation (Villetti et al., 2015).

In mice transiently transfected with the luciferase gene under the control of an NF-κB responsive element (NF-κB-luc) instillation of LPS in the lung induced an increase of bioluminescence imaging signal (Stellari et al., 2019). Tanimilast, administered by the snout-only inhalation route as a dry powder, decreased luciferase signal, cell airway infiltration and pro-inflammatory cytokine concentrations in BALF (Stellari et al., 2019).

Overall, tanimilast showed robust anti-inflammatory effects in various experimental models of pulmonary inflammation, in line with the anti-inflammatory effects observed *in vitro*. This is likely due to the high on target potency of tanimilast coupled with excellent lung retention. Indeed, tanimilast, administered intratracheally, resulted in dose-related, sustained concentrations of the compound in lung tissue and exhibited very limited systemic bioavailability, with plasma concentrations several orders of magnitudes lower than lung concentrations (Villetti et al., 2015). Accordingly, there were limited systemic effects upon inhalation observed in a study by Villetti et al. (2015), where tanimilast was ineffective in reversing ketamine/xylazineinduced anesthesia (a surrogate of emesis in rat) at a dose >50-fold higher than the anti-inflammatory ED50 observed in rats.

This finding was further strengthened by the observation that tanimilast, when given topically to ferrets (a relevant animal model for emesis/nausea), did not elicit emesis and nausea up to 10–20 mmol/kg, respectively whereas the PDE4 inhibitor GSK256066 (discontinued upon phase 2 clinical studies likely for safety issues) induced nausea at 1 mmol/kg intratracheally (Villetti et al., 2015). In summary, tanimilast was effective and well tolerated upon topical administration in pulmonary inflammation animal models relevant to COPD and asthma. Importantly, the therapeutic index of tanimilast upon inhaled delivery is excellent in preclinical animal models, with a robust anti-inflammatory activity coupled with minimal induction of systemic side effects typically associated with oral PDE4 inhibitors as well as with less optimized inhaled PDE4 inhibitors.

## Clinical Overview

### Phase I in Healthy Volunteers

The pharmacokinetic (PK) profile of tanimilast in healthy volunteers was investigated as single ascending doses (SAD) and multiple ascending doses (MAD) in two randomized, double-blind, placebo-controlled studies. The first in human (FIH) study (Table 3) was performed using a capsule-based single-dose dry-powder inhaler (DPI, Aerolizer^®^); the maximum doses tested were 2000 µg for the single dose and 1,600 µg once daily (OD) for 7 consecutive days for the repeated dose. The second study (FIH EXTENSION) (Table 3) was an extension of the FIH in which single (up to 4,800 µg) and repeated doses (up to 2,400 µg twice daily (BID); total daily dose of 4,800 µg) for 14 consecutive days were administered *via* the reservoir-based multi-dose DPI (NEXThaler^®^) (Mariotti et al., 2018). Tanimilast showed a linear dose-concentration pharmacokinetic profile. A modelling analysis demonstrated that the PK data was well described by a model in which parameters have no dose and time dependency (Jolling et al., 2018). This was a two-compartment disposition model with three-way parallel absorption pathways (slow, intermediate and fast) and first-order route of elimination. Physiologically, being the gastrointestinal drug availability of tanimilast very low (Villetti et al., 2015), the three absorption pathways may reflect three different compartments of the lung; a fast absorption pathway associated to the distal small airways and an intermediate and slow pathway associated to upper airway regions accounting for more than 60% of the absorbed dose (Jolling et al., 2018). The NEXThaler device in comparison to the Aerolizer provided a more efficient pulmonary drug deposition with ~30% higher drug availability. In addition, the BID versus the OD dosing regimen resulted in 35% lower fluctuation at steady state leading to a more stable concentration-time profile and potentially tighter target engagement at the receptor level throughout the dosing period (Mariotti et al., 2018). This was supported by an early physiologically based pharmacokinetic modelling study in which the BID regimen provided a receptor occupancy of >80% throughout 12 h for the 1,600 µg/day dose.

**TABLE 3:** Phase I studies.

Study ID short title	Main objectives	Study design	Device, dosing regimen, duration	Treatments (number of subjects)	Population (N randomized)	Primary measure(s)	References
Healthy volunteer							
First in human (FIH) study	Safety, tolerability and pharmacokinetics of single ascending doses (SAD) and multiple ascending doses (MAD) of tanimilast	SAD part: randomized, double-blind, placebo-controlled, single-dose escalation, alternating cross-over	Aerolizer^®^ single dose	Tanimilast: Dose 1: 20 µg (*n* = 8)	*N* = 20 Healthy subjects	Safety variables: AEs, ADRs, vital signs, 12-lead ECGs, 24 h ECG Holter monitoring, lung function, routine laboratory values, Pharmacokinetics variables	Mariotti et al. (2018)
				Dose 2: 100 µg (*n* = 8)			
				Dose 3: 200 µg (*n* = 8)			
				Dose 4: 400 µg (*n* = 7)			
				Dose 5: 800 µg (*n* = 7)			
				Dose 6: 1,600 µg (*n* = 7)			
				Dose 7: 2,000 µg (*n* = 6)			
				Matching Placebo (*n* = 14)			
		MAD part: randomized, double-blind, placebo-controlled, parallel group, multiple dose escalation	Aerolizer^®^ once-daily (OD), 7 days	Tanimilast: Dose 1: 100 µg OD (*n* = 7)	N = 54 Healthy subjects		
				Dose 2: 300 µg OD (*n* = 8)			
				Dose 3: 600 µg OD (*n* = 9)			
				Dose 4: 1,200 µg OD (*n* = 9)			
				Dose 5: 1,600 µg OD (*n* = 6)			
				Matching Placebo OD (*n* = 12)			
Main outcomes; Single ascending doses (SAD): All doses were well tolerated with no relevant gastrointestinal adverse effects. The Maximum Tolerated Dose (MTD) was not reached. Systemic exposure was linear and dose proportional in the range of doses evaluated. Multiple ascending doses (MAD): All doses were tolerated with no relevant gastrointestinal adverse effects. The MTD was not reached. Time to peak concentration (Tmax) in plasma was reached around 2–3 h post-dose, steady state was reached after 7–8 days consistent with an apparent half-life of around 40 h. Metabolite systemic exposure suggested limited formation *in-vivo*. The accumulation ratio after repeated OD dosing was around 2 to 3							
Healthy volunteer							
FIH Extension	Safety, tolerability and pharmacokinetics of SAD and MAD of tanimilast	SAD part: randomized, double-blind, placebo-controlled, single-dose escalation, alternating crossover	NEXThaler^®^ single dose	Tanimilast: Dose 1: 2,400 µg (*n* = 9)	*N* = 12 Healthy subjects	Safety variables: AEs, ADRs, vital signs, 12-lead ECGs, 24 h ECG Holter monitoring, lung function, routine laboratory values, Pharmacokinetics variables	Mariotti et al. (2018)
				Dose 2: 4,000 µg (*n* = 8)			
				Dose 3: 4,800 µg (*N* = 8)			
				Matching Placebo (*n* = 9)			
		MAD part: randomized, double-blind, placebo-controlled, parallel group, multiple dose escalation	NEXThaler^®^ Twice-daily (BID), 14 days	Tanimilast: Dose 1: 1,200 µg BID (*n* = 9)	*N* = 36 Healthy subjects		
				Dose 2: 2000 µg BID (*n* = 9)			
				Dose 3: 2,400 µg BID (*n* = 9)			
				Matching Placebo BID (*n* = 9)			
Main outcomes; Single ascending doses (SAD): All doses were tolerated with no relevant gastrointestinal or cardiovascular adverse effects. Time to peak concentration (T_max_) in plasma was reached around 3 h postdose. Mean half-life ranged from 40 to 49 h. Multiple ascending doses (MAD): All doses were well tolerated with no relevant gastrointestinal or cardiovascular adverse effects. The MTD was not reached. Systemic exposure was linear and dose proportional between 1,200 μg BID and 2,400 μg BID. T_max_ in plasma was reached around 1.5–3 h, steady state was reached after 7–8 days of treatment. The accumulation ratio after repeated BID dosing was around 6 to 7. Metabolites systemic exposure suggested limited formation *in-vivo*.							

### Phase IIa in Asthma

The first proof of mechanism for tanimilast was conducted in corticosteroid naïve mild allergic asthma patients who demonstrated a late asthmatic response (LAR) to inhaled allergen. The ability of tanimilast to reduce allergen challenge responses was investigated in a double blind, placebo controlled, 3-way cross-over study (ALLERGEN CHALLENGE) (Table 4) at two active dose levels; 400 and 1,200 µg OD for 9 consecutive days administered via the Aerolizer device prior to the challenge (Singh et al., 2016). The primary objective of the study was met with both doses of tanimilast significantly attenuating the LAR; 19.7% (*p* = 0.015) and 28.2% (*p* < 0.001) reduction for the lower and higher dose, respectively, of the area under curve (AUC) between 4 and 10 h post-challenge of the FEV_1_ percent changes (FEV_1_ AUC_4–10h_). Similar results were obtained using the absolute FEV_1_ AUC_4–10h_ changes and the maximum percent and absolute FEV_1_ fall compared to placebo. An important component of the LAR is an influx of inflammatory eosinophils into the airways (Imaoka et al., 2011). In this regard, tanimilast showed a numerical reduction of sputum eosinophils (30 and 42%, for the lower and higher dose, respectively) measured in a subset of subjects at 10 h post challenge (Singh et al., 2016). Notably, considering the ~30% higher drug availability of NEXThaler in comparison to Aerolizer (Mariotti et al., 2018), the doses used in this study were pharmacokinetically at least 3 to 10-fold lower than the highest dose currently under investigation in the Phase III program (1,600 μg BID; total daily dose of 3,200 μg) in COPD patients (PILASTER, 2020; PILLAR, 2020).

**TABLE 4:** Phase IIa studies.

Study ID short title	Main objectives	Study design	Device, dosing regimen, duration	Treatments (number of subjects)	Population (N randomized)	Primary measure(s)	References
Asthma							
Allergene challenge	Effect on induced airway response to allergen challenge in mild asthmatic patients, safety, tolerability and pharmacokinetics	Randomized, double-blind, placebo-controlled, three-way crossover	Aerolizer^®^ Once-daily (OD), 9 days	Tanimilast: Dose 1: 400 µg OD (*n* = 34)	*N* = 36 Mild asthma patients	Efficacy variables: Late asthmatic response (LAR), Safety variables: AEs, ADRs, vital signs, 12-lead ECGs, 24 h ECG Holter monitoring, routine laboratory values), Pharmacokinetics variables	Singh et al. (2016)
				Dose 2: 1,200 µg OD (*n* = 33)			
				Matching Placebo OD (*n* = 35)			
Main outcomes; Efficacy: Significant reduction in late asthmatic response (% change in FEV_1_ AUC_4-10_ post allergen challenge) compared to placebo (400 µg: −19.7%, 1,200 µg: −28.2%). Safety: All doses were well tolerated. The most frequently reported adverse events were headaches (400 µg: 20.6%, 1,200 µg: 12.1%, placebo: 11%). Dyspepsia was reported in 2.9% of patients on 400 µg dose. No other gastrointestinal adverse effects were reported. Pharmacokinetics: Time to peak concentration (T_max_) in plasma was reached around 2 h after dosing. Systemic exposure to tanimilast appeared dose proportional. Metabolites systemic exposure suggested limited formation *in-vivo*							
COPD							
Biomarker	Biomarkers of inflammation, safety and tolerability, pharmacokinetics	Multicenter, randomized, double blind, placebo-controlled, repeated dose, 3-way crossover	NEXThaler^®^ Twice-daily (BID) 32 days	Tanimilast: Dose 1:800 µg BID (*n* = 56)	*N* = 61 moderate or severe COPD patients with chronic bronchitis on maintenance with triple therapy (ICS, LABA and LAMA)	Pharmacodynamic variables: Biomarker of inflammation in blood and sputum, Safety variables: AEs, ADRs, vital signs, 12-lead ECGs, routine laboratory values, Pharmacokinetics variables	Singh et al. (2019), Singh et al. (2020b), Singh et al. (2020d); Govoni et al. (2020)
				Dose 2: 1,600 µg BID (*n* = 57)			
				Matching placebo BID (*n* = 57)			
Main outcomes; Pharmacodynamics: Significant reduction of a large number of mediators (both at protein and gene expression levels) associated to airway inflammation in comparison to placebo (including systemic level of SP-D) with limited systemic pharmacodynamic effect. Dose-response relationship both in terms of number of genes differentially expressed in sputum and in terms of fold change effect size of the differentially expressed genes. Post hoc analyses in patients with higher eosinophils counts (≥3% in sputum) showed a significant effect of tanimilast on the reduction of eosinophils and other key type-2 mediators in sputum. Safety: All doses were well tolerated. The most common AEs were nasopharyngitis (24.6%), reported with a comparable incidence for all treatments including placebo, and headache (11.5%). The incidence of gastrointestinal adverse events (e.g. diarrhea, nausea, vomiting, abdominal pain) was low without dose relationship. Pharmacokinetics: Exposure to tanimilast as well as to metabolites was broadly proportional with doses. Sputum levels of tanimilast at 2 h post-dose at steady state were approximately 2000-fold higher than the corresponding levels in plasma, with dose proportionality							

The results of this study were consistent with those obtained with roflumilast in asthma patients where a significant attenuation of LAR and other mediators was also observed (Bardin et al., 2015). This suggests that PDE4 inhibitors might have potential applications in respiratory conditions characterized by a type-2 inflammatory component, including asthma. Further studies on lung function, symptoms and exacerbations are deemed necessary to confirm a role of tanimilast in this therapeutic area.

### Phase IIa in Chronic Obstructive Pulmonary Disease

**FIGURE 2:**
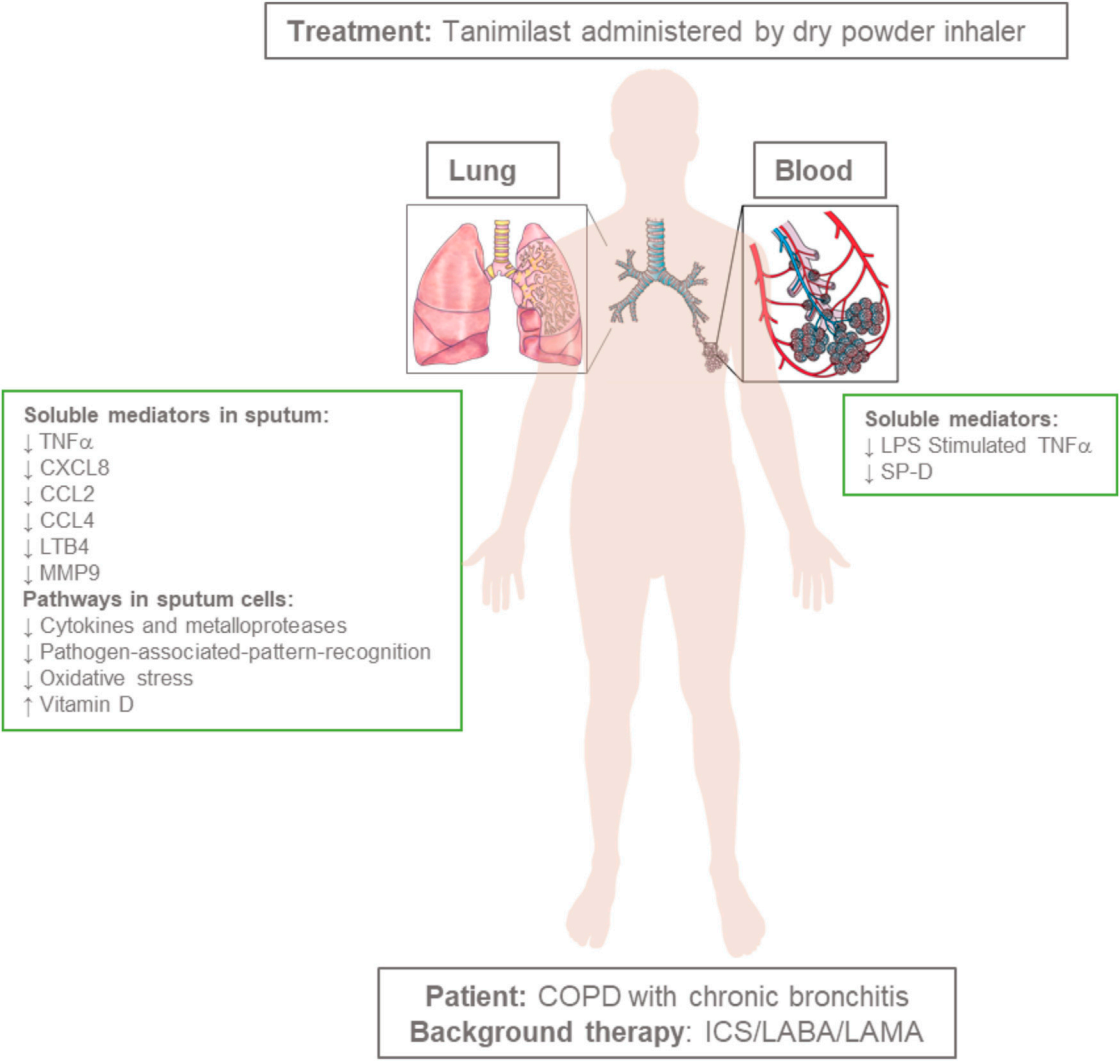
Administration of tanimilast (total daily doses of 1,600 or 3,200 μg) to COPD patients with a chronic bronchitis phenotype on top of inhaled triple therapy (ICS, LABA and LAMA) results in a broad anti-inflammatory profile in the airways associated with limited systemic effects. Figure objects were adapted by RexxS, Public domain, DataBase Center for Life Science (DBCLS), CC BY 4.0 <https://creativecommons.org/licenses/by/4.0>, and Artwork by Holly Fischer, CC BY 3.0 <https://creativecommons.org/licenses/by/3.0>, via Wikimedia Commons.

The anti-inflammatory profile of tanimilast was characterized in COPD patients in a three-period, three-way, placebo-controlled, double-blind, complete-block crossover study (BIOMARKER) (Figure 2 and Table 4) in which patients with a chronic bronchitis phenotype received tanimilast doses of 800 or 1,600 μg BID (total daily doses of 1,600 or 3,200 μg) or matching placebo on top of inhaled triple therapy (ICS, LABA and LAMA), all via the NEXThaler device for 32 consecutive days (Singh et al., 2019). Sixty-one COPD patients (mean age 66; 65% current smokers) with mean post-bronchodilator FEV_1_ 50.2% predicted and mean COPD Assessment Test (CAT) score of 20.7 were randomized in the study.

The systemic exposure to tanimilast was dose proportional and comparable to that observed in healthy volunteers in the FIH EXTENSION study (Table 3) using the same inhaler device (NEXThaler) (Mariotti et al., 2018). Metabolic profiling in the systemic circulation highlighted the rapid formation of two pharmacologically less active metabolites (Armani et al., 2014; Cenacchi et al., 2015) generated by tanimilast hydrolysis whose concentrations were 20 and 50-fold lower than that of the parent compound. Notably, the exposure in sputum measured by a validated mass spectrometry method (Singh et al., 2019) was approximately 2000-fold higher than that in the systemic circulation for both doses. Accordingly, the pharmacodynamic profile of tanimilast highlighted a major effect in sputum but not in blood. After 32 days of treatment both doses of tanimilast significantly reduced inflammatory mediators in sputum in comparison to placebo such as leukotriene B4 (LTB4), C-X-C motif chemokine ligand 8 (CXCL8), macrophage inflammatory protein 1β (MIP1β or CCL4), matrix metalloproteinase 9 (MMP9), and tumor necrosis factor α (TNFα). Monocyte chemotactic protein 1 (MCP-1 or CCL2) was also significantly reduced at the 800 μg BID dose. These are all pro-inflammatory mediators playing a key role in the pathophysiology of COPD; CXCL8 and LTB4 acting as neutrophil chemoattractants (Pease and Sabroe, 2002; Woolhouse et al., 2002), TNFα causing amplification of inflammation (Barnes, 2016), MMP-9 being a protease involved in airways remodeling (Beeh et al., 2003; Churg et al., 2012), CCL2 and CCL4 activating C-C chemokine receptor type 2 (CCR2) and type 5 (CCR5) and contributing to inflammatory cell recruitment (Deshmane et al., 2009; Costa et al., 2016).

The relative effect of tanimilast on sputum inflammatory cells showed a numerical reduction of eosinophils, lymphocytes and macrophages by a 10–30% in comparison to placebo both in terms of absolute and percent values. This effect was not observed on neutrophils.

At variance from sputum, there were no major pharmacodynamic effects of the treatment in the systemic compartment in terms of suppression of the inflammatory cytokines in serum. However, stimulation of TNFα production by an *ex vivo* challenge of blood samples with lipopolysaccharide (LPS) (blood incubated with 30 µL of LPS 5 µg/ml for 23 h) was significantly inhibited with 1,600 μg BID. This whole blood *ex-vivo* model may mimic the systemic immune response during an exacerbation. These results suggest that the low systemic exposure in stable COPD conditions did not exert detectable anti-inflammatory effects, but that upon interaction with pathogenic material (LPS *ex-vivo* model) circulating drug levels were sufficient to limit the inflammatory response.

Notably, both doses of tanimilast significantly reduced concentrations of the surfactant protein-D (SP-D) in blood. This protein is primarily expressed in pulmonary non-ciliated bronchiolar epithelial cells (Crouch et al., 1992) and has a pleiotropic role being involved in anti-inflammatory, anti-microbial, and innate immune defense processes (Sorensen, 2018). Loss of air–blood barrier integrity by disease-induced lung injury might favour the outward intravascular leakage of proteins, allowing SP-D produced in the airways to leak into the bloodstream. Increased SP-D blood levels, often associated with decreased concentrations in the airways, were shown both in preclinical and clinical inflammatory lung conditions (Honda et al., 1995; Fujita et al., 2005; Winkler et al., 2011; Gaunsbaek et al., 2013; Moazed et al., 2016). Relevance of these SP-D variations and their association with clinical outcomes were observed in cystic fibrosis (Noah et al., 2003; Olesen et al., 2010), COPD (Sin et al., 2007; Sims et al., 2008; Lomas et al., 2009; Winkler et al., 2011) and asthma (Mackay et al., 2016).

The biological effect of tanimilast observed at protein level was validated and further characterized at transcriptional level in a post-hoc whole-genome gene expression microarray analysis in whole blood and sputum cells (Govoni et al., 2020). In line with the results for inflammatory proteins measured in the serum, the impact of tanimilast on gene expression in blood was minimal with either dose. In contrast, in sputum, a large number of probe-sets were significantly impacted after 32 days of treatment; the highest dose having the greatest effect with 1,471 and 2,598 significantly differentially-expressed probe-sets relative to placebo (p-adjusted for False Discovery Rate (FDR) < 0.05) with 800 and 1,600 μg BID, respectively. Further, there was a straightforward consistency between dose; 84% of the probe sets significantly differentially expressed with the lowest dose were also significantly differentially expressed with the highest dose with all common genes following the same directionality (i.e., either both up regulated or both down regulated) and with a clear dose-response relationship also in terms of fold change effect size between doses. Functional enrichment analysis in sputum cells highlighted a clear trend toward decreased inflammatory conditions with a significant modulation of key COPD pathways involved in cytokine activity, pathogen-associated-pattern-recognition activity, oxidative stress and vitamin D with associated inhibition of downstream inflammatory effectors (Govoni et al., 2020). Cytokines and matrix metalloproteinases play a major role in the regulation of immune response and inflammatory conditions (Srivastava, et al., 2007; Barnes, 2008). Analysis of the genes significantly differentially expressed (pFDR <0.05) and coding for cytokines and matrix metalloproteinases clearly showed that the vast majority of these mediators were downregulated for both doses including macrophage inflammatory protein-1-alpha (CCL3) and CCL4, interleukin-27-beta (IL-27B), interleukin-12-beta (IL12B), interleukin-32 (IL32), tumor necrosis factor alpha induced protein-8 (TNFAIP8), ligand-superfamily member-15 (TNFSF15), and matrix metalloproteinases (MMP)-7,12 and 14. Downregulation of IL-12, IL-32 and IL-27 and MMPs confirmed a potential role of tanimilast on type-1 inflammation and lung remodeling, both playing key roles in the pathophysiology of COPD (Saetta et al., 2002; Barnes, 2008; Calabrese et al., 2008; Cao et al., 2012; Churg et al., 2012). Consistent with observations at the protein level, downregulation of CCL3 and CCL4 corroborated the central role of the drug on CCR5 mediated inflammatory and chemoattractant signaling (Barnes, 2008; Costa et al., 2016). Notably, the gene coding for CCR5 and its ligand CCL5 (RANTES proteins) were both significantly downregulated at both dose levels. A significant effect of tanimilast was also observed on type-2 inflammation; both doses led to a negative regulation of Th2 cytokine production [upregulation of SCGB1A1 and TNFRSF21 (Mandal et al., 2004; Benschop et al., 2009; UniProt, 2021)], B cells activation/proliferation [upregulation of SAMSN1 and INPP5D (Zhu et al., 2004; Akerlund et al., 2015; UniProt, 2021)] and eosinophils and basophils activation [downregulation of PRG2, CLC, OLIG1, OLIG2 and PRSS33 (George and Brightling, 2016; Metcalfe et al., 2016; Sridhar et al., 2019; UniProt, 2021)].

Gene expression analysis showed consistency with findings at target protein levels, allowing further characterization of the pharmacodynamic profile of tanimilast, showing a broad anti-inflammatory and modulatory effect of the drug in the airways of patients already on treatment with triple therapy (ICS, LABA, and LAMA). Despite the strong pharmacodynamic effect in the lung, tanimilast did not improve FEV1 significantly after 32 days of treatment.

### Phase IIb in Chronic Obstructive Pulmonary Disease

The efficacy of tanimilast in COPD patients was investigated in a 24-weeks, randomized, double-blind, double-dummy placebo- and active-controlled, parallel-group, dose-ranging Ph2b study (PIONEER) (Singh et al., 2020c) (Table 5). At study entry, symptomatic patients with a history of at least one moderate or severe exacerbation in the previous year and using stable maintenance therapy with an ICS and a LABA were switched to LABA monotherapy (formoterol fumarate 12 μg BID) for 2 weeks prior to randomization. At the baseline visit, patients received one of the six following treatments on top of formoterol fumarate 12 μg BID: one of four tanimilast doses (400, 800, 1,200 or 1,600 μg BID) via the NEXThaler device, budesonide 400 μg BID *via* a different DPI, or placebo.

**TABLE 5:** Phase IIb study.

Study ID short title	Main objectives	Study design	Device, dosing regimen, duration	Treatments (number of subjects)	Population (N randomized)	Primary measure(s)	References
COPD							
PIONEER	Efficacy, safety, and tolerability	Multicenter, randomized, double-blind, double-dummy, placebo- and active-controlled, parallel group, dose ranging	NEXThaler^®^ Twice-daily (BID) 24 weeks	Tanimilast: Dose 1: 400 µg BID	N = 1,130 moderate or severe COPD patients with a history of exacerbation on maintenance with LABA therapy	Primary efficacy variable: Predose FEV_1_ at week 12, Main secondary efficacy variables: COPD exacerbation, PROs, Blood biomarkers, Safety variables: AEs, ADRs, vital signs, 12-lead ECGs, routine laboratory values	Singh et al. (2020c)
				Dose 2: 800 µg BID			
				Dose 3: 1,200 µg BID			
				Dose 4: 1,600 µg BID			
				Budesonide DPI			
				400 µg BID			
				Matched placebo BID			
Main outcomes; Efficacy: No difference versus placebo were observed for any dose of tanimilast or for the active control budesonide on pre-dose FEV_1_ at 12 and 24 weeks or on PROs. Clinically relevant reductions in the COPD exacerbation rate (moderate or severe) were observed with all the tanimilast doses on top of LABA compared to placebo (LABA alone); from 13 to 28% for the highest dose. A post hoc analysis on subgroup populations showed an enriched effect in terms of exacerbation rate reduction in the subgroups of patients with chronic bronchitis; this effect increased further in patient with chronic bronchitis and blood eosinophils ≥150 cells/μL reaching statistically significance for the 800 and 1,600 μg BID doses. A significant reduction of systemic level of SP-D for all doses of tanimilast (but not for budesonide) was observed in comparison with placebo. Safety: All the doses well tolerated. The incidence of known PDE4 inhibitor class effects (e.g., gastrointestinal) was low and comparable to that of the placebo group							

1130 COPD patients (mean age 64; 53% current smokers) with mean post-bronchodilator FEV_1_ of 48.1% predicted, mean CAT score of 20.5 and mean modified Medical Research Council dyspnea scale (mMRC) score of 2.3 were randomized. 56% had a chronic bronchitis phenotype and the remainders had emphysema or a mixed phenotype. Study outcomes highlighted a lack of effect on the lung function endpoints for any of the active treatments (including budesonide as positive control) compared to placebo. In addition, there was no consistent treatment–placebo or tanimilast–budesonide differences in any of the symptom-related endpoints. Notably, there were marked improvements from baseline in all groups including placebo, with mean changes from baseline being close to, or exceeding, clinical relevance for Transition Dyspnea Index (TDI; 1 unit), St. George’s Respiratory Questionnaire (SGRQ; 4 units) and Evaluating Respiratory Symptoms (E-RS; 2 units). Previous studies have shown a significant effect of budesonide plus formoterol compared with formoterol alone both on pulmonary function and symptoms (Calverley et al., 2003; Tashkin et al., 2008; Rennard et al., 2009; Ferguson et al., 2017). Therefore, the lack of effect observed in this trial with budesonide treatment used as positive control suggested that a “trial effect” occurred. Such a trial effect can arise due to improved medical care (compared to pre-trial) being administered to all participants; this may explain the lack of difference for active treatments versus placebo on lung function and patient reported outcomes measuring symptoms and quality of life. Additionally, these patients reported outcomes often show improvements in placebo arms due to the subjective nature of symptom reporting coupled with the double blinding involved in clinical trials. Differently from lung function and symptoms, exacerbation rates did not appear to be influenced by a trial effect; budesonide showed a significant reduction (39%) in the annualized rate of exacerbations compared to placebo (*p* = 0.030). Numerical exacerbation rate reductions were observed also with the four tanimilast treatment groups compared with placebo, ranging from 13 to 28% for the highest dose. An effect of tanimilast was also observed on blood biomarkers with a significant reduction of SP-D levels in comparison to placebo both at week 12 and week 24. Notably this effect was not observed in the budesonide group and was consistent with the results of the BIOMARKER study (Table 4) where tanimilast was given in COPD patients with chronic bronchitis on top of triple therapy, indicating a specific effect of inhaled PDE4 inhibition irrespective of the study population and the background therapy (Singh et al., 2019; 2020c). The impact of tanimilast on SP-D might indicate a relevant pharmacological effect associated with prevention of COPD deterioration, although this needs to be confirmed in larger, longer studies.

### Post-Hoc Studies in Subtype Populations

In the phase IIb PIONEER study (Table 5) the relative effect on exacerbation rate reduction of tanimilast versus placebo was numerically larger in patients with a chronic bronchitis phenotype (from 24 to 37%) or in patients with baseline blood eosinophil count ≥150 cells/μL (ranging from 17 to 51%; statistically significant (*p* < 0.05) for 1,600 µg BID dose). When both chronic bronchitis and blood eosinophils ≥150 cells/μL where combined together, the reduction of the exacerbation rate increased further from 49 to 73% (statistically significant for the 800 and 1,600 μg BID doses). These post-hoc analyses indicated that chronic bronchitis and higher eosinophil counts are determinants of response for tanimilast in COPD and generated hypotheses to be prospectively validated in the phase III clinical program and further trials.

The effect of tanimilast on exacerbation rate reduction in the subgroup of chronic bronchitis patients with higher blood eosinophil counts is consistent with previous results with the oral PDE4 inhibitor roflumilast. Post-hoc, pooled analyses of phase III roflumilast studies showed a reduction of exacerbation frequency in a subset of COPD patients whose characteristics included chronic bronchitis with or without concurrent use of ICS (Rennard et al., 2011). These observations led to the design of subsequent pivotal studies that prospectively confirmed a reduction in exacerbation rates mediated by roflumilast given on top of ICS and long acting bronchodilators in COPD patients with a chronic bronchitis phenotype and a history of exacerbations (Martinez et al., 2015). Furthermore, post-hoc analyses of two randomized controlled trials (Martinez et al., 2015, 2016) demonstrated an association between higher blood eosinophil counts and greater effects of roflumilast on exacerbation prevention (Martinez et al., 2018). Notably, this enhanced effect was observed in patients with higher eosinophils counts on top of ICS and bronchodilators, which is a population known to be responsive to ICS (Pizzichini et al., 1998; Agusti et al., 2018; Siddiqui et al., 2018; Singh et al., 2020a; GOLD, 2021).

The mechanisms responsible for these differential drug effects is unknown but may relate to an increased presence of type-2 inflammation in COPD patients with higher blood eosinophil counts causing different responses to anti-inflammatory drugs (Higham et al., 2020). In a recent post-hoc analysis of the BIOMARKER study (Table 4) focused on sputum gene expression it was shown that patients with chronic bronchitis and higher sputum eosinophil counts on treatment with triple therapy had increased expression of type-2 and PDE4 related genes (Singh et al., 2020b). In this investigation a large number of genes were differentially expressed (*p*-FDR<0.05) between patients having different sputum eosinophil level (<3% or ≥3%); all genes were upregulated in patients with higher eosinophil counts and functionally enriched for type-2 and PDE4 inflammatory processes including interleukin 5 receptor alpha (IL5RA), interleukin 4 (IL4), chemokine (C-C motif) ligand 26 (CCL26), arachidonate 15-lipoxygenase (ALOX15), Interleukin 1-receptor-like 1 (IL1RL1), charcot-Leyden crystal galectin (CLC), GATA-binding protein 1 (GATA1), sphingomyelin phosphodiesterase 3 (SMPD3), cysteinyl leukotriene receptor 2 (CYSLTR2), prostaglandin D2 receptor 2 (PTGDR2) and CCAAT enhancer binding protein epsilon (CEBPE) and the cAMP-specific PDE4 isoform D (PDE4D). Mechanistically these findings indicate that despite the concomitant use of ICS, COPD patients with higher eosinophilic counts display a specific profile of type-2 and PDE4 related airway inflammation that may be targeted with PDE4 inhibitors. In line with these observations the ability of tanimilast to reduce eosinophils and other key type-2 mediators in sputum (e.g. IL5RA, CLC, ALOX15, SMPD3, PTGDR2, and CEBPE) was most relevant to individuals with higher sputum eosinophil counts (≥3%) (Singh et al., 2020d; 2020b). These data are in line with the reduction of sputum and bronchial mucosal eosinophils observed in patients treated with roflumilast (Rabe et al., 2018) and support the rationale for PDE4 inhibition on top of inhaled corticosteroids and bronchodilators also in patients with higher eosinophil counts.

In this post-hoc analysis of the BIOMARKER study (Table 4) eosinophilic COPD patients were defined using sputum eosinophil counts while most of the previous associations between clinical effect and eosinophilia were done using blood counts. Diverging results have been published so far on the relationship between blood and sputum eosinophils studies (Eltboli and Brightling, 2013; Singh et al., 2014; Negewo et al., 2016; Schleich et al., 2016; Hastie et al., 2017; Rabe et al., 2018; Tashkin and Wechsler, 2018; Pignatti et al., 2019). However, in this cohort of patients there was a good predictability of blood eosinophils to identify sputum eosinophilia. Receiver operating characteristic (ROC) curves analysis showed that both per cent and absolute blood eosinophils were able to predict patients with sputum eosinophil level <3% or ≥3% with area under the curves (AUCs) of 0.82 and 0.79, respectively (*p* < 0.001), with good correlation between percent sputum and blood eosinophils (Pearson r ≥ 0.46, *p* ≤ 0.0002) (Singh et al., 2020d).

### Phase III in Chronic Obstructive Pulmonary Disease

COPD patients who are still symptomatic and continue to exacerbate despite receiving inhaled triple therapy treatment account for approximately 30–40% (Vestbo et al., 2017; Langham et al., 2019). As of today these patients have limited or no additional treatment options (GOLD, 2021). On the basis of such a high unmet clinical need and of the determinants of response identified during phase II, tanimilast is progressing into the next stages of clinical development as a treatment to reduce the risk of exacerbations in patients with COPD associated with chronic bronchitis and a history of exacerbations, as an add-on to a combination of ICS, a LABA, and a LAMA. The efficacy and safety of two tanimilast doses (800 and 1,600 μg BID) administered *via* the NEXThaler device for 52 weeks is being assessed in this specific population in two randomized, double-blind, placebo-controlled phase III studies (PILASTER; PILLAR) (Table 6). The primary outcome measure is the rate of moderate and severe COPD exacerbations occurring during the planned 52-weeks treatment period. Eligible patients are required to have at least one moderate or severe COPD exacerbation in the previous year. In the PILASTER study 2,985 patients with post-bronchodilator FEV1 <80% of the subject predicted normal value are planned to be enrolled. In the PILLAR study active-control with roflumilast as a comparator arm (250 µg once daily during the first 4 weeks followed by 500 µg once daily for the remaining treatment period) is included in the study in a double-dummy fashion. In this study 3,980 patients with a higher severity of lung function impairment (post-bronchodilator FEV1 <50% of predicted) are planned to be enrolled.

**TABLE 6:** Ongoing Phase III studies.

Study ID short title	Main objectives	Study design	Device, dosing regimen, duration	Treatments (number of subjects)	Population (N randomized)	Primary measure(s)	References
COPD							
PILASTER	Efficacy, safety, and tolerability	Multicenter, Randomized, Double-blind, Placebo-controlled, Parallel-group	NEXThaler^®^ Twice-daily (BID) 52 weeks	Tanimilast: Dose 1: 800 µg BID	*N* = 2,985 moderate to very severe COPD patients with chronic bronchitis and a history of exacerbation on maintenance with triple therapy (ICS, LABA and LAMA)	Primary efficacy variable: Rate of moderate and severe exacerbations, Main secondary variables: Time to 1st moderate or severe exacerbations, predose FEV_1,_PROs, Safety variables: AEs, AEs of special interests, vital signs, body weight, 12-lead ECGs, routine laboratory values	(PILASTER)
				Dose 2: 1,600 µg BID			
				Matched placebo BID			
PILLAR	Efficacy, safety, and tolerability	Multicenter, Randomized, Double-blind, Double-dummy, Active and placebo-controlled, Parallel-group	NEXThaler^®^ Twice-daily (BID) 52 weeks	Tanimilast: Dose 1: 800 µg BID	*N* = 3,980 severe to very severe COPD patients with chronic bronchitis and a history of exacerbation on maintenance with triple therapy (ICS, LABA and LAMA)	Primary efficacy variable: Rate of moderate and severe exacerbations, Main secondary variables: Time to 1st moderate or severe exacerbations, predose FEV_1,_ PROs, Safety variables: AEs, AEs of special interests, vital signs, body weight, 12-lead ECGs, routine laboratory values	(PILLAR)
				Dose 2: 1,600 µg BID			
				Roflumilast			
				500 µg OD			
				Matched placebo			

### Safety

The main class effects of PDE4 inhibitors include GI and other related side effects as diarrhea, nausea, decreased appetite, weight loss and psychiatric conditions (Muñoz-Esquerre et al., 2015; Rogliani et al., 2016). For this reason, the use of the oral PDE4 inhibitor roflumilast is dampened by its safety and tolerability profile. This has prompted the search for inhaled PDE4 inhibitors to maximize the therapeutic effect in the lung and minimize the systemic events possibly leading to treatment discontinuation (Phillips, 2020). Tanimilast was shown to provide drug levels in the lung ~2000-fold higher than those observed in the systemic circulation. Approximately 1,000 subjects were exposed to tanimilast up to the completion of phase II clinical program. Overall, the drug was well tolerated, with no evidence of PDE4 inhibitors class-related side effects as GI events and weight loss leading to treatment discontinuation.

Studies in healthy volunteers (FIH and FIH EXTENSION) (Table 3) showed no relationship between tanimilast dose and the occurrence of adverse events (AEs) or serious adverse events (SAEs). AE including those potentially related to treatment were similarly distributed between treatments including placebo. There were no significant mean laboratory values outside normal range, no clinically significant post-dose decreases in FEV_1_ (bronchospasm) and no clinically significant cardiovascular events.

In asthmatic patients (ALLERGENE CHALLENGE study) (Table 4) and COPD patients with chronic bronchitis (BIOMARKER study) (Table 4) a similar proportion of adverse events was reported, including those potentially related to the treatment, for either dose of tanimilast and placebo. Adverse events were mostly mild in nature, the most common being headache in asthmatics and nasopharyngitis in COPD. There were no major differences between treatments (including placebo) in any of the hematology or biochemistry data as well as for cardiovascular parameters.

In the largest study conducted in COPD patients (PIONEER study; *n* = 1,130) (Table 5) all treatments (four different doses of tanimilast, placebo and budesonide as comparator) were well tolerated with similar distribution of AEs across treatment groups and with no tanimilast dose-effect. The incidence of class effect AEs or pneumonia was low, and similar in all groups (including placebo and budesonide). A total 72 events, reported in 47 subjects (4.2%), were considered as potentially treatment related. None of them were serious or severe with no trends for differences across treatments. There were five deaths; one subject each in the tanimilast 400 μg BID, 800 μg BID, and 1,200 μg BID groups and two in the tanimilast 1,600 μg BID group; none of them were considered treatment related. There were no treatment-related trends in biochemistry, hematology and urinalysis and no clinically significant mean changes from baseline in cardiovascular parameters or in bodyweight.

An in-depth evaluation of the effects of tanimilast on cardiac repolarization and cardiac arrhythmias was conducted in healthy volunteers using repeated 24-h electrocardiogram recordings from the FIH and FIH EXTENSION studies (Table 3). The global cardiac safety profile of tanimilast showed the absence of potential meaningful QTc liability as well as PR and QRS prolongation or change in heart rate or cardiac arrhythmia counts (Ferrari et al., 2016). In particular, concentration–QT data were analyzed by means of mixed-effects modelling (Jolling et al., 2019). The slope of the concentration-QTcF relationship was not significantly greater than 0 and the simulations showed that the upper limit of the 90% confidence interval around the mean ΔΔQTcF (baseline and placebo corrected QTcF) was not expected to exceed 10 milliseconds within the range of clinically relevant concentrations. In this model extrapolated 10 milliseconds limit was predicted to be reached at a concentration of ~18,000 pg/ml, much higher than the highest concentration (12,700 pg/ml) and the geometric mean Cmax (~5,700 pg/ml) observed after multiple twice-daily dosing of 2,400 μg BID in the EXTENSION study (Table 4). The outcome of these analyses suggested that tanimilast is unlikely to significantly prolong the QT interval at a clinically relevant dose.

## Conclusion

Tanimilast was selected among a series of novel benzoic ester derivatives for its potent and selective inhibitory activity versus PDE4 combined with ADME and pharmacokinetics properties rendering it suitable for inhaled administration. Tanimilast proved to be highly potent and effective in inhibiting the release of several inflammatory mediators from different clinically relevant human cellular preparations, including, peripheral blood mononuclear cells, lymphocytes, macrophages, eosinophils, neutrophils, dendritic cells and airways epithelial cells. Preclinical studies conducted with tanimilast in different rodent models of acute and sub-chronic pulmonary inflammation triggered by different stimuli, ranging from bacterial endotoxins, to allergens and cigarette smoke, showed robust anti-inflammatory effects. In these *in vivo* experiments tanimilast was delivered to the airways at doses resulting in sustained concentrations in lung tissue and scarce systemic exposure. This translated in limited systemic effects as assessed in two distinct experimental models (rat and ferret) for emesis (Villetti et al., 2015), a typical on target side effect of PDE4 inhibitors.

In line with the preclinical findings, clinical investigations showed a broad anti-inflammatory profile of tanimilast in the airways associated with limited systemic effects; this was consistent with an exposure of the drug in sputum approximately two thousand-fold higher than that in blood and with no meaningful evidence of class-related side events (Singh et al., 2019; 2020c). Consistently with observations with the oral PDE4 inhibitor roflumilast in COPD, the chronic bronchitis phenotype was found to be a determinant of response in terms of exacerbation rate reduction (Singh et al., 2020c). In patients with chronic bronchitis, tanimilast administered on top of ICS, a LABA and a LAMA consistently reduced a large number of key mediators associated to airways inflammation, remodeling and lung integrity (Singh et al., 2019; Govoni et al., 2020). Notably, patients treated with triple therapy and having higher eosinophil counts at baseline (and thus patients known to respond better to ICS) showed increased levels of sputum cell inflammation associated to type-2 and PDE4 mediators (Singh et al., 2020b). These findings supported the potential for tanimilast to provide an additional beneficial effect in patients with chronic bronchitis who are still symptomatic despite regular use of ICS, LABA and LAMA, with a favorable systemic tolerability profile. The role of tanimilast in this specific population is currently under investigation in two large randomized, double-blind, placebo-controlled phase III pivotal trials (PILASTER; PILLAR) (Table 6).

Population analyses in asthmatics and COPD patients with higher eosinophilic counts showed a potential role of tanimilast also in conditions characterized by a relevant type-2 inflammatory component. In asthma, tanimilast significantly attenuated the drop in FEV_1_ induced by an allergen challenge in the late allergen reaction phase which is characterized by a large influx of inflammatory eosinophils into the airways (Singh et al., 2016). In COPD, subtype population analyses in patients with chronic bronchitis and higher eosinophils counts showed a significant effect of tanimilast on the reduction of eosinophils and other key type-2 mediators in sputum (Singh et al., 2020d; 2020b). Finally, in patients with chronic bronchitis and blood eosinophils ≥150 cells/μL tanimilast significantly reduced the exacerbation rate at the doses selected for Phase III clinical development (Singh et al., 2020c). These biological and clinical data coupled together indicate an effect of tanimilast on type-2 inflammation which could translate in meaningful clinical outcomes. Further studies are deemed necessary to confirm a role of tanimilast in specific populations characterized by type-2 inflammatory features.
